# Supplementary material for: Changes in Cartilage Thickness and Denuded Bone Area after Knee Joint Distraction and High Tibial Osteotomy—Post-Hoc Analyses of Two Randomized Controlled Trials
Source: J Clin Med. 2021 Jan 19;10(2):368. doi: 10.3390/jcm10020368 (PMC7835945; doi:10.3390/jcm10020368)
Supplement: Supplementary file 1 [file jcm-10-00368-s001.pdf]

1. Longitudinal Changes by Patient Group: Subregions

Cartilage thickness changes in the sixteen subregions are shown in Figure S1 for each of the three groups. The KJD_{HTO} group did not show statistically significant thickness changes in any of the regions, except for a small but statistically significant decrease in the internal LAC tibia. The HTO group showed a statistically significant cartilage thickness decrease in the central tibia, internal tibia, and internal femoral areas of the MAC and internal femoral area of the LAC, while the external tibial LAC area showed a statistically significant increase. The KJD_{TKA} group showed a significant increase in cartilage thickness in the central, anterior, and external areas of the tibia and femur of the MAC, while the internal and posterior areas of the MAC and all areas in the LAC showed no statistically significant change over time.

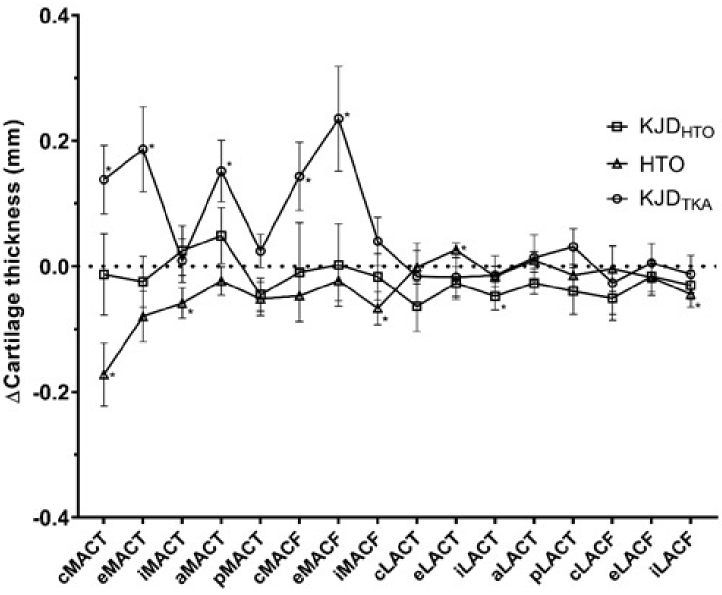

Figure S1. Two-year cartilage thickness changes in each of the 16 subregions. Subregions are the central (c), external (e), internal (i), anterior (a) and posterior (p) parts of the tibia (T) and the central, external and internal parts of the femur (F) for both the most (MAC) and least (LAC) affected compartment. KJD_{HTO} = KJD patients from the high tibial osteotomy (HTO) trial; KJD_{TKA} = knee joint distraction (KJD) patients from the total knee arthroplasty trial. Significant two-year changes are indicated with *. Means and standard errors are shown.

2. Longitudinal changes by patient group: location-independent results

The location-independent cartilage thickening scores were 0.81±0.93 mm for KJD_{HTO}, 0.55±0.48 mm for HTO, and 1.62±0.95 mm for KJD_{TKA}. The thinning scores were −1.14±0.95 for KJD_{HTO}, −1.14±1.48 for HTO, and −0.72±0.69 for KJD_{TKA}. Kruskal-Wallis tests showed no statistically significant difference between the three groups in thinning scores ($p = 0.23$) but the thickening score was significantly greater for KJD_{TKA} than for the other two groups as confirmed by post-hoc Dunn tests (KJD_{HTO} $p = 0.016$; HTO $p = 0.001$). Yet, this was no longer true for the comparison KJD_{TKA} *vs* KJD_{HTO} when correcting for significantly different baseline characteristics using linear regression ($p = 0.505$).

3. Prediction of cartilage thickness changes

A multivariable linear regression model, using patient baseline characteristics and baseline MAC cartilage thickness as independent variables, revealed that only the Kellgren-Lawrence grade was a significant predictor (B = 0.105; p= 0.01) of MAC cartilage thickness change in all KJD and HTO patients together: a higher Kellgren-Lawrence grade was associated with in a greater increase in cartilage thickness during treatment.

Using treatment as independent variable resulted in a better fit of the univariable model ($R^2 = 0.120$ with $p = 0.004$ compared to $R^2 = 0.095$ with $p = 0.01$), therefore KJD and HTO treated patients were evaluated in separate models as well. For HTO-patients, none of the parameters, including leg axis deviation, significantly predicted the MAC cartilage thickness or JSW change. In contrast, in KJD patients a multivariable linear regression model left only Kellgren-Lawrence grade as a significant predictor for MAC cartilage thickness change.

Univariable models showed that patient age ($B = 0.018$; $R^2 = 0.128$; $p = 0.04$), baseline MAC cartilage thickness ($B = -0.165$; $R^2 = 0.207$; $p = 0.006$) and patient group KJD_{TKA}/KJD_{HTO} ($B = -0.245$; $R^2 = 0.133$; $p = 0.03$) significantly predicted cartilage change as well, but Kellgren-Lawrence grade ($B = 0.174$; $R^2 = 0.255$; $p = 0.002$) remained the strongest predictor.

4. Longitudinal changes by baseline severity: sensitivity analyses

Table S1. Sensitivity analyses for comparisons where patients were included in different trials.

Parameter	KJD_{mild} *vs* KJD_{severe}		KJD_{mild} *vs* HTO_{mild}		KJD_{severe} *vs* HTO_{severe}	
	**Difference**	***p***	**Difference**	***p***	**Difference**	***p***
MAC ThCtAB (mm)	0.42 (0.15–0.68)	**0.003**	0.09 (−0.08–0.27)	0.289	−0.31 (−0.59–0.03)	**0.031**
MAC dABp (%)	−5.9 (−13.8–2.0)	0.139	−1.4 (−4.5–1.7)	0.351	7.5 (−0.8–15.9)	0.075
Mean MAC JSW	0.61 (−0.35–−1.57)	0.204	0.48 (−0.03–1.00)	0.064	−0.16 (−1.11–0.80)	0.742

KJD_{mild} = knee joint distraction (KJD) patients with mild osteoarthritis (OA); KJD_{severe} = KJD patients with severe OA; HTO_{mild} = high tibial osteotomy patients with mild OA; HTO_{severe} = HTO patients with severe OA; MAC = most affected compartment; ThCtAB = mean cartilage thickness over the total subchondral bone area (in mm); dABp = percentage of denuded subchondral bone area; JSW = joint space width; Difference = mean difference between groups corrected for statistically significant between-group differences and trial in which patients were included. Differences are shown with mean and 95% confidence interval. Bold *p*-values indicate statistical significance ($p < 0.05$), calculated with linear regression, correcting for statistically significantly different baseline characteristics and trial.

4. Supplementary discussion

In KJD patients, the anterior region of the MAC tibia and the central and external regions of the MAC tibia and femur clearly showed the most substantial cartilage restoration. The baseline cartilage thickness in the central femur and external tibia and femur was much smaller than that of the other regions (≥40%; data not shown). This could explain the greater restoration in these three areas. In another MRI cartilage study, the anterior tibial region has been shown to be frequently involved in both thickening and thinning of cartilage [36]. Similarly, in another study, the central tibial and femoral regions showed a greater loss of cartilage than the other regions [37]. As such, the higher rate of cartilage restoration at the central, anterior, and external parts of the MAC may be the result of natural sensitivity to change.
